# Characterizing the amyloid core region of the tumor suppressor protein p16^INK4a^ using a limited proteolysis and peptide-based approach

**DOI:** 10.1016/j.jbc.2024.107590

**Published:** 2024-07-18

**Authors:** Sarah G. Heath, Jennifer D. Naughton, Nicholas J. Magon, Shelby G. Gray, Briana R. Smith, Vanessa K. Morris, Christoph Göbl

**Affiliations:** 1 Mātai Hāora - Centre for Redox Biology and Medicine, Department of Pathology and Biomedical Science, University of Otago, Christchurch, New Zealand; 2 School of Biological Sciences, University of Canterbury, Christchurch, New Zealand; 3 Biomolecular Interaction Centre, University of Canterbury, Christchurch, New Zealand

**Keywords:** amyloid, protein stability, protein oxidation, p16^INK4a^, mass spectrometry

## Abstract

The human tumor suppressor p16^INK4a^ is a small monomeric protein that can form amyloid structures. Formation of p16^INK4a^ amyloid fibrils is induced by oxidation which creates an intermolecular disulfide bond. The conversion into amyloid is associated with a change from an all α-helical structure into β-sheet fibrils. Currently, structural insights into p16^INK4a^ amyloid fibrils are lacking. Here, we investigate the amyloid-forming regions of this tumor suppressor using isotope-labeling limited-digestion mass spectrometry analysis. We discover two key regions that likely form the structured core of the amyloid. Further investigations using thioflavin-T fluorescence assays, electron microscopy, and solution nuclear magnetic resonance spectroscopy of shorter peptide regions confirm the self-assembly of the identified sequences that include methionine and leucine repeat regions. This work describes a simple approach for studying protein motifs involved in the conversion of monomeric species into aggregated fibril structures. It provides insight into the polypeptide sequence underlying the core structure of amyloid p16^INK4a^ formed after a unique oxidation-driven structural transition.

The tumor suppressor protein p16^INK4a^ (hereafter referred to as p16) is an all α-helical, monomeric protein that tightly binds to cyclin-dependent kinases 4 and 6 (CDK4/6), thereby inhibiting cell cycle progression from the G1 to S phase (1, 2). We recently reported that p16 folds into amyloid fibrils when its single cysteine residue is oxidized (3). The otherwise stable and monomeric protein can form an intermolecular disulfide bond to create a homodimer intermediate that subsequently assembles into extended amyloid structures. The short-lived dimeric species is partially α-helical and has a lower melting temperature compared to the monomer (4). p16 is the first example of solely oxidation-induced amyloid formation, and the first protein with an ankyrin-repeat fold known to convert into amyloid (3). We recently identified the presence of p16 dimers and likely amyloids in cancer cells without addition of exogenous oxidant, suggesting the presence of oxidized p16 in cells (4). Our previous data show that the amyloid state of p16 is not able to inhibit CDK4/6 and therefore potentially represents a loss-of-function state. It is currently unclear which residues contribute to this dramatic structural change from all α-helical into β-sheet.

Here, we characterized the monomeric and amyloid structure of p16 using a limited proteolysis approach (5). In this method, analysis of peptides using mass spectrometry reveals different peptide peak intensities over time depending on the surface accessibility and flexibility surrounding the protease cleavage sites, allowing conclusions to be drawn about the molecular structures. This approach has provided valuable insights into the structural assembly of other amyloids. For example, limited digestion of amyloid-β suggested that it has a flexible N-terminus, which converts into amyloids from a mainly intrinsically disordered state (6). Amyloids with structured precursor proteins have also been characterized through this approach; the all α-helical protein BAX is converted to fibrils through interaction with the peptide humanin, and it was shown that large regions of BAX are protease-inaccessible in the BAX-humanin amyloids (7).

For the characterization of p16 amyloids, we used heavy and light nitrogen isotope-labeled versions of p16 with simultaneous processing to minimize experimental variation. We identified two protected regions of p16 amyloids. The identified fragments were studied in isolation using fluorescence-based amyloid formation assays, solution nuclear magnetic resonance (NMR) spectroscopy, and electron microscopy. Peptide experiments of the regions around the sole cysteine residue of p16 suggest that a triple-methionine region, a tetra-leucine region, and the cysteine neighbors greatly drive the transition into amyloid, with potential contributions of a hydrophobic stretch of residues 91 to 99. Alignment of the human p16 sequence with homologs of other organisms identified the presence of these key motifs around the largely conserved cysteine residue. This suggests that the transition of p16 into amyloid could be a general feature within this family of tumor suppressor proteins and it could have a functional role.

## Results

### Cysteine oxidation converts monomeric p16 into amyloids

**Figure 1:**
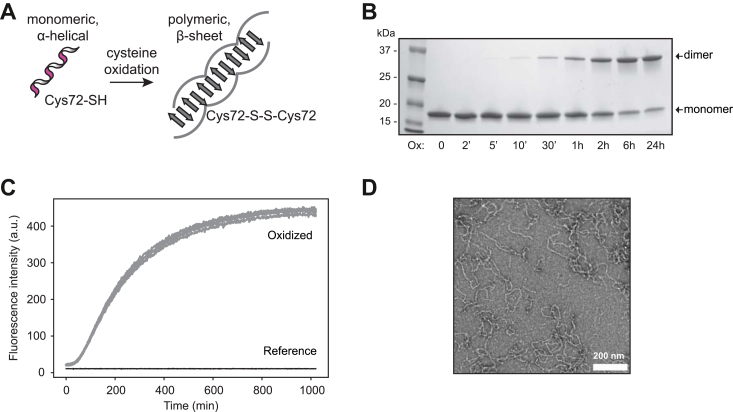
**Oxidation-induced amyloid formation of p16.***A*, proposed mechanism of p16 amyloid formation driven by cysteine oxidation (3). *B*, oxidation of p16 (20 μM) with diamide (200 μM) triggers disulfide-dependent dimerization as analyzed by non-reducing SDS-PAGE. A representative gel image is shown from at least three experiments. *C*, under the same conditions, thioflavin-T fluorescence analysis tracks the structural conversion of p16 to amyloid following oxidant exposure (*gray*, 200 μM diamide), whereas unoxidized protein is not converted to amyloid (*black*). Analysis was performed in quadruplicate. *D*, p16 amyloid morphology is visible on negative-stained transmission electron micrographs; image acquired after 24 h oxidation of 20 μM p16 treated with 200 μM diamide. The micrograph is representative of three experiments.

We recently reported that oxidation leads to amyloid formation of p16 (3). The transition involves disulfide-bond formation of its single cysteine residue to create a homodimeric species that subsequently folds into amyloids (Fig. 1, *A* and *B*). The standard experimental conditions used here were 20 μM protein solution incubated with 200 μM oxidant. We used tetramethylazodicarboxamide (commonly referred to as diamide) as the amyloid-converting oxidant. This compound is frequently used for its specificity to induce disulfide bonds, and it does not cause unspecific oxidation events that would modify the molecular weights of the digested peptides (8). To determine amyloid formation kinetics, we performed thioflavin-T (ThT) fluorescence analysis and found that amyloid formation plateaus at about 8 h (Fig. 1*C*). The amyloids formed were stable and showed typical dimensions observed for amyloid fibrils by electron microscopy (Fig. 1*D*) as reported earlier (3).

### Digestion of p16 and peptide identification

**Figure 2:**
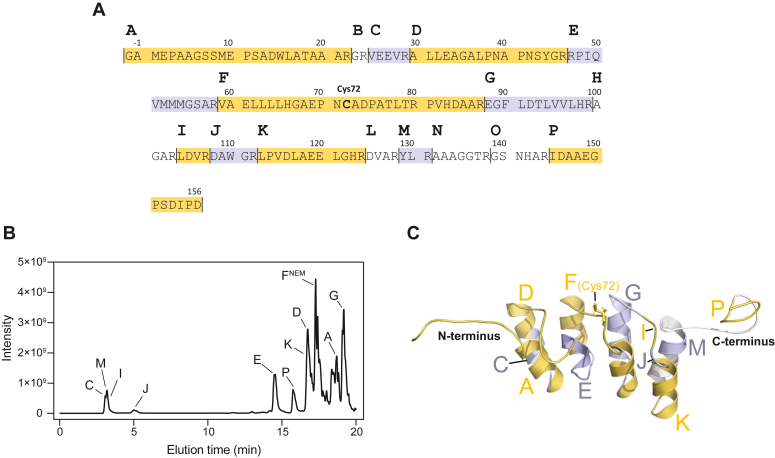
**Full tryptic digestion of p16.***A*, depiction of theoretical peptides upon full trypsin digestion (labeled A–P). Experimentally detected tryptic peptides are highlighted in alternating *yellow* and *gray* to illustrate sequence coverage. *B*, a single representative total ion chromatogram (m/z 300–2000) after trypsin digestion of monomeric p16 for 24 h, with peptide elution times indicated. F^NEM^ represents the alkylated form of peptide F after treatment with 10 mM NEM for 15 min. *C*, a three-dimensional structure of p16 (PDB 2A5E (11)) with tryptic peptides labeled. NEM, *N*-ethylmaleimide.

We first digested native monomeric p16 with the protease trypsin at 37 °C, which preferentially cleaves after arginine and lysine residues (9, 10). The hypothetical tryptic peptides of p16 (labeled alphabetically A–P) are illustrated in Figure 2*A* and are visualized on the three-dimensional structure of the monomeric conformation in Figure 2*C*. The monomeric protein is most stable in low salt buffer systems (11) and therefore a 4 mM Hepes solution at pH 7.4 was used during the protease digestion steps. Before addition of trypsin, p16 samples were incubated with 10 mM *N*-ethylmaleimide (NEM) to block free cysteine thiols and prevent formation of artifacts during digestion and analysis. Using ThT fluorescence assays, we confirmed that this alkylation step does not induce amyloid formation of monomeric protein. The monomeric protein was digested to completion for 24 h and analyzed by mass spectrometry, resulting in the identification of a number of tryptic peptides representing >85% sequence coverage (Fig. 2, *A* and *B* and Table S1). We concluded that trypsin yields reproducible digestion throughout the sequence in triple-replicated experiments.

### Time-dependent digestion of monomeric and amyloid p16 shows different accessibility patterns of released peptides

Although multiple structures of monomeric p16 have been determined in the unbound state (11) or in complex with binding partners (12), it is a protein with a remarkably low melting temperature (13). We determined the melting temperature to be 42.9 ± 0.3 °C using dynamic scanning fluorimetry (Fig. S1*A*). Because we aimed for a structure-dependent digestion process unbiased by temperature instability, we tested digestion of p16 at the lower temperature of 25 °C, which yielded the same cleavage products as observed previously at 37 °C.

We then optimized two additional parameters impacting time-dependent digestion: the protease-to-substrate ratio and the digestion time. Protein digestion using trypsin-to-p16 weight ratios of 1:10, 1:50, and 1:100 for 30 min yielded the best reproducibility, and we therefore applied a 100-fold excess of p16 per trypsin molecule for the subsequent time series experiments. To determine the digestion times, monomeric p16 was incubated using the described conditions for 5, 15, 30, 45, 60, 90, and 120 min. Full digestion was observed after 30 min. We then tested the same digestion conditions on amyloid p16. For this, p16 was first incubated with the oxidant diamide for 16 h prior to digestion with trypsin. When comparing the extent of digestion between monomeric and amyloid p16, differences in peptide peak areas were mainly observed after shorter trypsin incubations.

In order to accurately quantify the differences between the monomeric and amyloid protein states, we expressed uniformly ^15^N-labeled protein by growing *Escherichia coli* in minimal medium with ^15^NH_4_Cl as the sole nitrogen source. This allowed us to combine different isotope versions of monomeric and amyloid protein in a single digestion and mass spectrometry analysis step, thereby reducing variations in digestion, sample injection, and peptide ionization. The resulting natural abundance isotope (hereafter referred to as ^14^N-labeled) and larger mass ^15^N-labeled peptides were then used to distinguish the two different protein conformations. The ^15^N-labeled protein had a similar melting temperature (44.0 ± 0.2 °C) to the unlabeled protein (42.9 ± 0.3 °C) (Fig. S1*A*) and also exhibited similar amyloid formation rates as shown by ThT fluorescence analysis (Fig. S1*B*). The HPLC elution times of the ^15^N-labeled peptides upon full digestion were essentially identical to the ^14^N-labeled variants (Fig. S1*C* and Table S1). We performed the experiments in duplicate, one in which ^14^N-labeled monomeric protein was combined with ^15^N-labeled amyloid p16 and the other using the alternate labeling scheme. Samples of amyloid and monomer p16 were combined and digested for 5, 15, 30, and 120 min before analysis by mass spectrometry.

**Figure 3:**
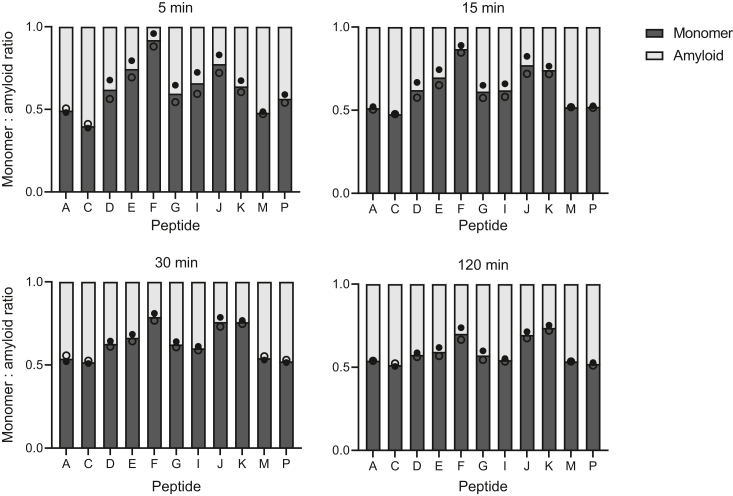
**Limited trypsin digestion of monomeric and amyloid p16 analyzed by mass spectrometry.** Monomer and amyloid samples of p16 were combined (each 10 μM) and digested at 25 °C for 5 to 120 min using 1:100 trypsin:protein weight ratio. The experiment was performed using ^14^N and ^15^N p16 for the monomeric and amyloid protein, respectively, and repeated *vice versa*. Amyloid protein was prepared by incubating 20 μM monomeric p16 with 200 μM diamide for 16 h at room temperature. Peptide peak areas of both ^14^N and ^15^N species were quantified and are plotted as a proportion of the total species. Duplicate data points (independent replicates) are displayed as open and filled dots. Peptides are labeled as presented in Fig. 2*A*. The data for the C72-containing peptide F are represented by the signal obtained for the alkylated form of peptide F after treatment with NEM in the monomer (F^NEM^) and for the disulfide-linked peptide (F-F) in the amyloid. NEM, *N*-ethylmaleimide.

The observed peak areas of peptides from both monomeric and amyloid protein (alternating ^14^N and ^15^N versions) were quantified and are displayed in Figure 3 as a fraction of the total peptide. We found that the relative abundance of peptides at shorter digestion times was greater in the monomeric protein sample, indicating higher accessibility of the trypsin cleavage sites than in the amyloid conformation. Over time, all peptide ratios converged toward an approximately 50:50 ratio (Fig. 3). The N- and C-terminal regions of the protein show similar ratios even at earlier time points. These regions contain intrinsically disordered sequences in the monomer, and these data suggest that they are also accessible in the amyloid state. Nevertheless, some peptides appear less accessible in the amyloid state based on their lower relative abundance. In particular, peptides E, F (the latter containing the cysteine residue), and J seem to be protected in the fibril conformation.

### Amyloid conversion of isolated p16 regions

**Figure 4:**
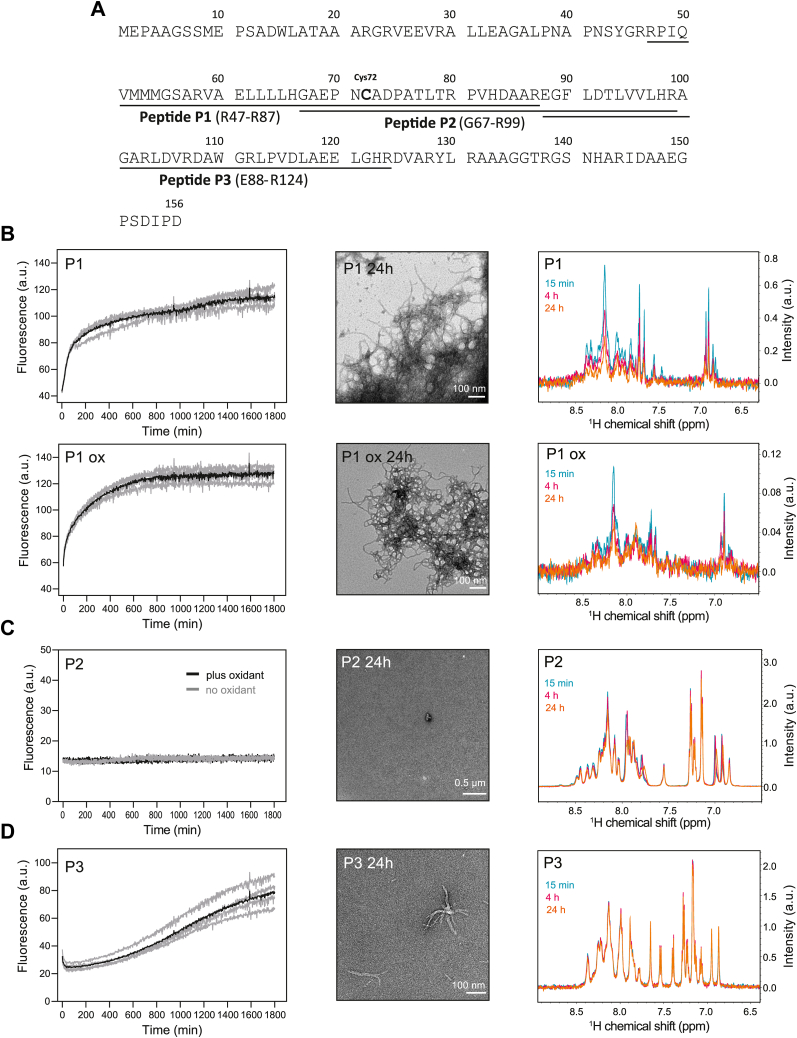
**Design of truncated regions of p16 and measurement of amyloidogenic properties.***A*, amino acid sequence of human p16 with peptide regions P1–P3 labeled. *B*–*D*, ThT amyloid assays (*left*), transmission electron micrographs (*middle*, n = 3), and ^1^H amide region solution NMR spectra (*right*, n = 1) for P1–P3. Samples of 80 μM peptide in 10 mM phosphate buffer pH 7.4 were made immediately prior to measurement. For ThT assays, individual replicates (4) are shown in *gray*, while the average is overlaid in *black* except in the case of P2 where replicates are plotted both for untreated (*gray*) and oxidized (*black*). Samples treated with oxidant (ox) had 200 μM diamide added immediately after dissolving the peptide. NMR, nuclear magnetic resonance; ThT, thioflavin-T.

Based on the mass spectrometry digestion data, we next designed three truncated versions of p16 (named P1–P3), of 33 to 41 amino acids in length. P1 spans peptides D to F, P3 spans peptides G to K, and P2 covers a central region with terminal overlap of P1 and P3. The individual sequences are mapped on the amino acid sequence of p16 in Figure 4*A*. These peptides were first tested for their propensity to form amyloid by conducting ThT fluorescence analysis. Both P1 and P2 harbor the reactive cysteine residue, and we performed the amyloid formation assays in the presence and absence of the cysteine-specific oxidant diamide. As displayed in Figure 4, *B* and *D*, the P1 and P3 regions generated an increase in ThT fluorescence over time, suggesting the formation of β-sheet–based amyloid structures, whereas P2 fluorescence stayed at baseline levels. Aggregation half-times were determined using Amylofit analysis software by taking the plateau at 800 min as the endpoint (14). The approximate half-time of P1 aggregation in the absence of oxidation was 72.6 ± 9.7 min, and the oxidized form yielded 127.4 ± 27.4 min, although the different curve shapes do not allow for direct comparison of kinetics.

In agreement with the results from ThT assays, transmission electron microscopy (TEM) micrographs of P1 showed extensive amyloid formation both in the presence and absence of oxidant following overnight incubation (Fig. 4*B*). Only small amorphous aggregates lacking fibrillar morphology were identified in peptide P2, also in agreement with ThT analysis (Fig. 4*C*). Amyloid fibrils were also visible in micrographs of P3, albeit to a lesser extent than for P1, possibly reflecting the difference in formation kinetics observed by ThT assays (Fig. 4*D*). Consistent with these findings, Fourier-transform infrared analysis of P1 showed an amide I peak maximum at 1624 cm^−^^1^, characteristic of cross β-sheet amyloid secondary structure, while Fourier-transform infrared spectra of P2 and P3 were inconclusive, showing broad peaks more characteristic of non-amyloid β-sheet or random coil (Fig. S3) (15, 16).

We further studied the self-assembly of P1–P3 using solution NMR spectroscopy. This method measures the monomeric peptide hydrogen signal intensities, whereas the large aggregates become undetectable. Individual samples were measured immediately upon dissolving and at later time intervals, and the resulting spectra are presented in Figure 4, *B*–*D*. P1 and oxidized P1 lost signal over time (quantified in Fig. S2), as did P3, which likely reflects their transition into larger species, in accordance with the ThT and TEM analysis. The signal intensity of P2 stayed constant over time.

### Identification of a short peptide region highly prone to amyloid formation

We then sought to specify the regions within P1 and P3 that were potentially forming amyloids. Several shorter peptides spanning P1 and P3 were selected based on features such as sequential hydrophobic residues. These peptides were named P1.1–P1.7 and P3.1–P3.4 (Fig.s 5*A* and 6*A*). As P1.4 had poor predicted solubility, the otherwise identical peptide P1.5 was designed with an additional C-terminal arginine residue (based on solubility prediction using pepcalc.com, Innovagen AB). These peptides were freshly dissolved and diluted to 80 μM for further analysis.

**Figure 5:**
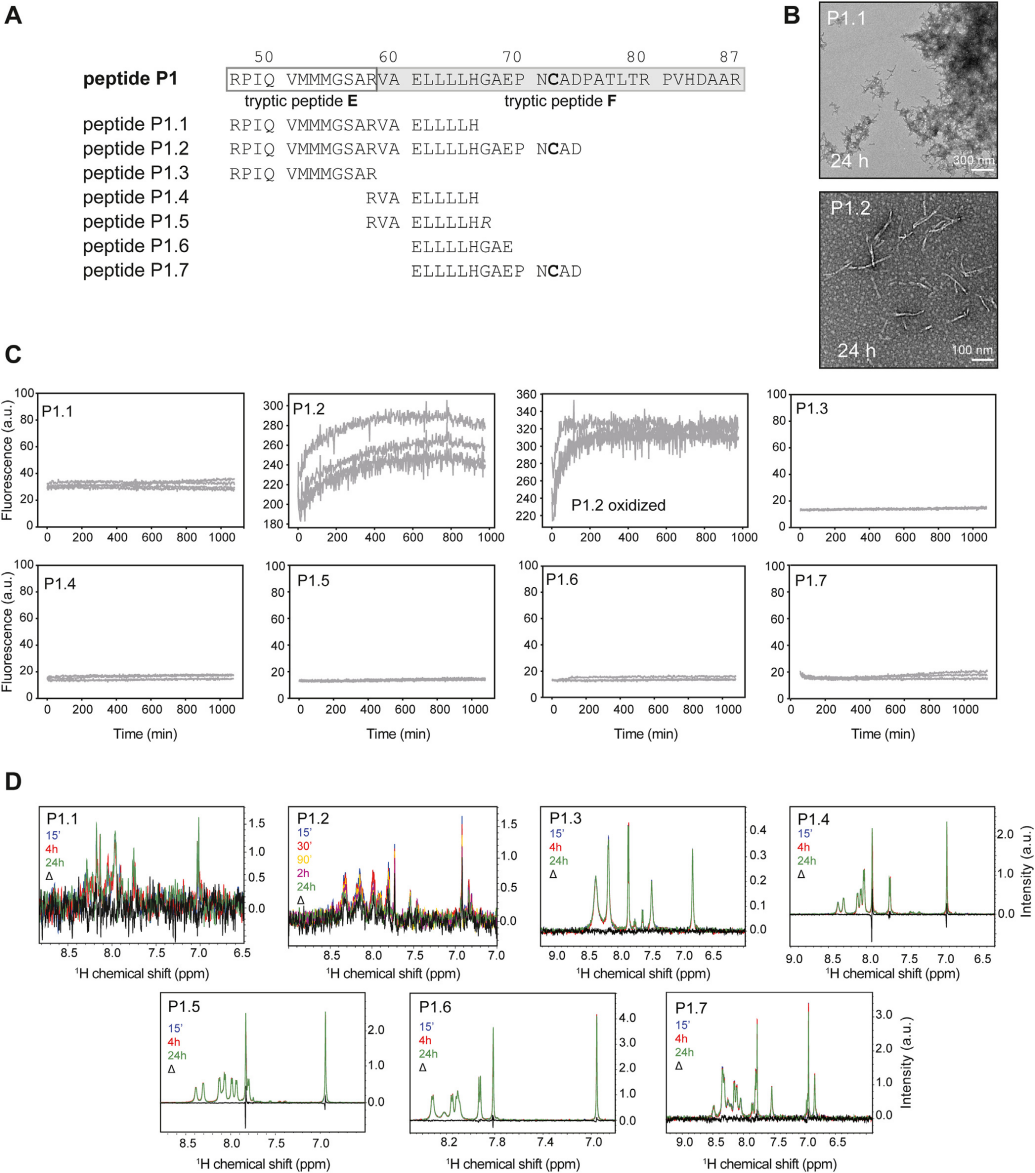
**Dissecting amyloid-forming region P1 to specify amyloidogenic sequence.***A*, peptides P1.1–P1.7 were designed to span the p16 truncated region P1 and the tryptic peptides E–F. *B*, transmission electron micrographs of 80 μM peptide samples P1.1 and P1.2 24 h after dissolving in a 10 mM phosphate buffer, pH 7.4. Representative images are shown from three experiments. *C*, ThT amyloid formation assays of P1.1–P1.7 performed in quadruplicate. The C72-containing peptide P1.2 was also measured in the presence of 200 μM diamide. *D*, ^1^H amide region solution NMR spectra. Peptide samples were dissolved as described and measured over a 24 h period (n = 1). *Black traces* (Δ) show the difference between the 15 min and 24 h spectra. NMR, nuclear magnetic resonance; ThT, thioflavin-T.

The peptides were measured using time-dependent ThT fluorescence analysis in the presence and absence of oxidant. Among the P1 series of peptides, only P1.2 gave an increase in ThT fluorescence, while the other peptides showed no increase either in the presence or absence of diamide (Fig. 5*C*). Electron microscopy measurements of the peptide P1.2 confirmed morphology typical of amyloid fibrils (Fig. 5*B*). The half-time of P1.2 amyloid formation in the absence of oxidant was 230 ± 37 min, and it was significantly faster at 25 ± 13 min in the presence of diamide, despite lacking sigmoidal curve shapes (14). We then performed solution NMR analysis on this peptide series. ^1^H NMR spectra were obtained at different time points after dissolving peptides, and only peptide P1.2 showed loss of signal intensity over time both in the absence and presence of oxidant, as would be expected during the process of aggregation and loss of free peptide (Fig. 5*D*). Peptide P1.1 showed aggregation during the NMR time series (Fig. 5*D*) and showed aggregates with possible fibrillar morphology in TEM (Fig. 5*B*); however, no ThT binding was detected for this peptide (Fig. 5*C*). It is possible that this peptide forms ThT-negative amyloid fibrils, which have been previously reported for other peptide fragments of amyloid proteins (17, 18, 19) and may be a consequence of the short length of the peptide or due to its overall morphology impairing ThT binding.

**Figure 6:**
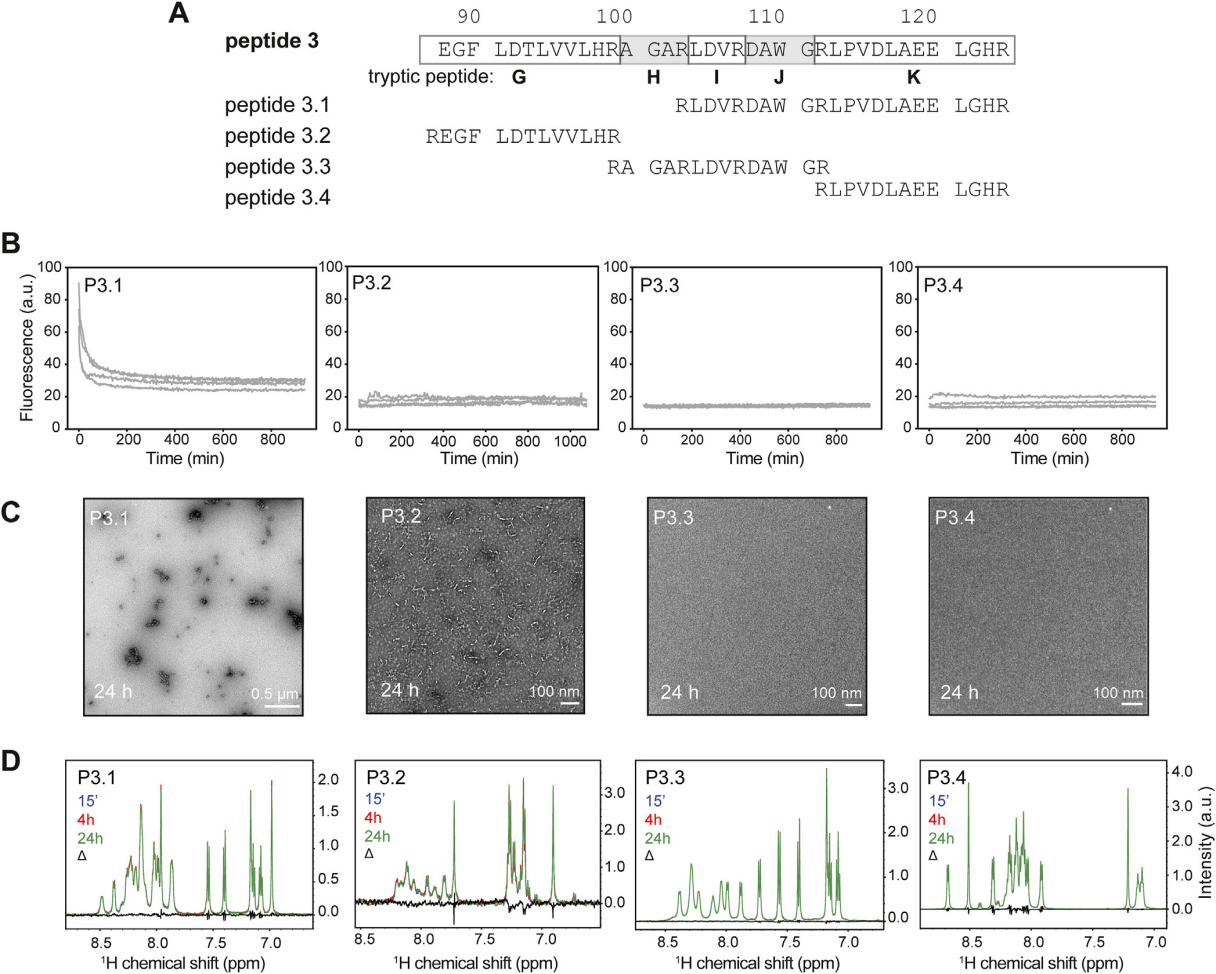
**Testing short peptide regions within P3 for their propensity to form amyloid.***A*, design of peptide series P3.1–P3.4 spanning the full-length P3 region and tryptic peptides G–K. *B*, thioflavin-T amyloid formation assays of 80 μM peptide in 10 mM phosphate buffer pH 7.4 performed in quadruplicate. *C*, negative-stain transmission electron microscopy analysis of 80 μM peptide solutions 24 h after incubation at room temperature. Images are representative of at least three experiments. *D*, ^1^H amide region solution NMR spectra. Peptide samples were dissolved as described for ThT and EM assays and measured over a 24 h period (n = 1). *Black traces* (Δ) represent the difference between the 15 min and 24 h spectra. NMR, nuclear magnetic resonance; ThT, thioflavin-T.

None of the P3 series of shorter peptides displayed changes in ThT fluorescence (Fig. 6, *A* and *B*) despite the signal increase given by the full length P3 region in ThT assays and the presence of some fibrils in electron microscopy (Fig. 4). Only peptide P3.1 displayed an initial drop in intensity (Fig. 6*B*), which may result from the presence of insoluble, amorphous aggregates, supported by the electron micrograph images (Fig. 6*C*). Despite the absence of ThT binding and no major detectable change in NMR signal intensity (Fig. 6*D*), electron microscopy revealed the presence of numerous short fibrils in the peptide P3.2 sample (Fig. 6*C*). Overall, these data highlight the major contribution of the sequence corresponding to P1.2 and a possible minor contribution of P3.2 in p16 amyloid fibril formation.

## Discussion

Oxidation of the single cysteine residue of the α-helical protein p16 leads to formation of a homodimeric species that subsequently folds into amyloid. The mechanism of this unusual, oxidant-induced conversion and the residues involved in the amyloid structure are currently unknown. Here, we identified key regions of the p16 protein sequence that are prone to aggregate into amyloid-type structures.

By performing limited digestion coupled to mass spectrometry analysis, we compared the protease accessibility of cleavage sites in the monomeric and the amyloid state. When incubating the protein with trypsin for various time points, we found that digestion times as low as 5 min yielded reproducible peptide fragments, and full digestion was observed at about 90 min for both the monomer and the amyloid. These short digestion times seem to reflect the low stability and high flexibility of the monomeric protein in particular and are comparable to a previous study that applied a similar approach to study p16 cancer-related mutations (20). While longer than for the monomer, the digestion times for the amyloid state were surprisingly short, suggesting that this conformation is also not particularly stable. We frequently observe that the fibrils can easily be dissolved in SDS buffer without heating (3), in contrast to many other amyloid structures (6, 21, 22, 23, 24).

The parallel digestion of the monomeric and amyloid species using ^14^N and ^15^N isotope labeling greatly improved the reproducibility and accuracy of the data and allowed for assignment of each peptide to its original structural state. We observed a clear trend where the monomeric protein yielded more peptides around the central region, whereas the termini were similarly accessible at early time points. This may suggest that the N- and C-termini are not part of the amyloid structural core, which is supported by their polar, glycine, and proline-rich amino acid composition. In contrast, peptide F showed the lowest digestion rates in the amyloid state. Experiments of P1 and P1.2, which both contain (parts of) peptide F, showed different ThT amyloid formation rates in the presence of oxidant, suggesting a strong role of the cysteine residue even for the shorter peptide versions.

**Figure 7:**
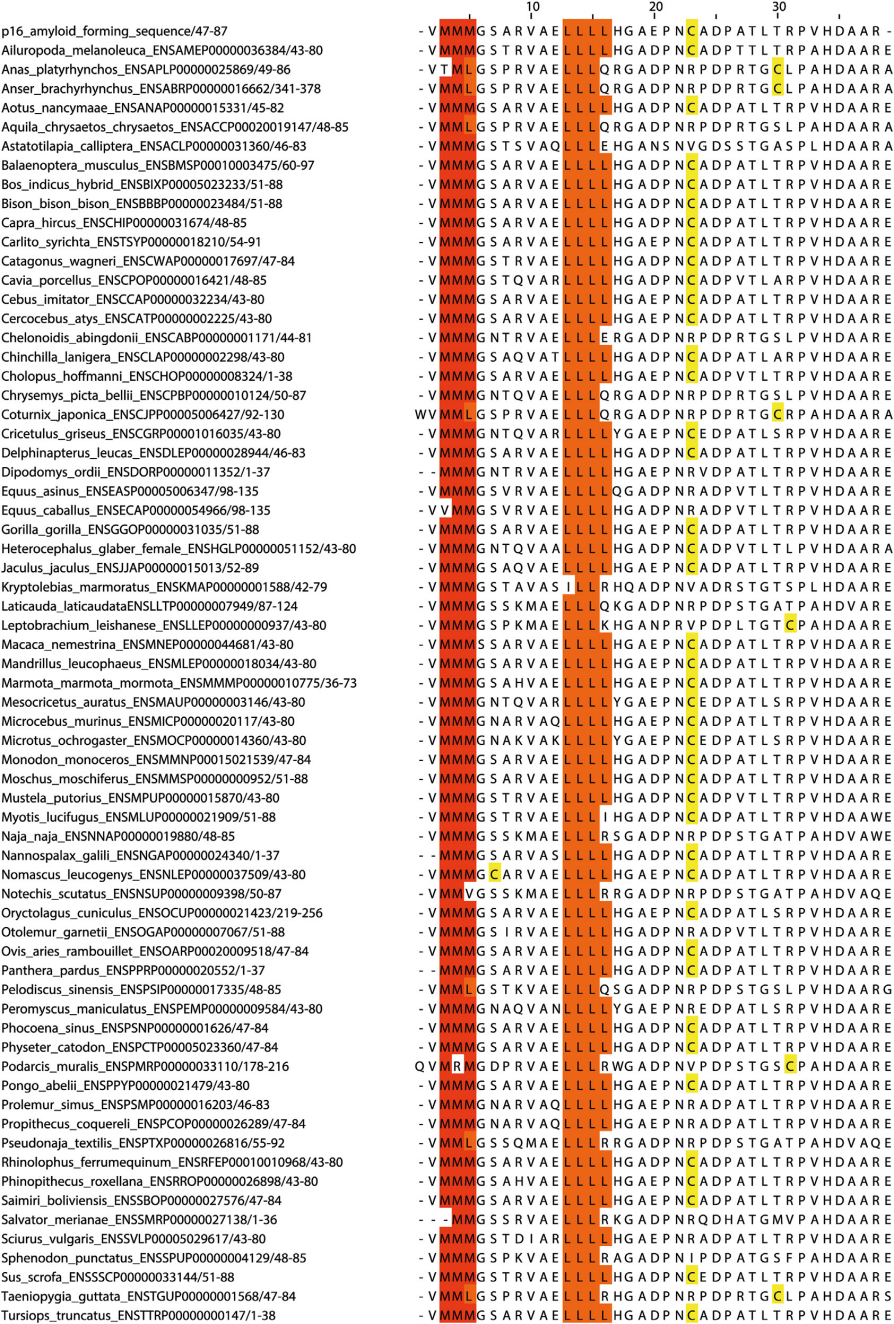
**Sequence conservation analysis of the amyloid-forming region of human p16 and of other vertebrate species.** Protein sequences are referred to by their ENSEMBL ID and conservation of methionine (*red*), leucine (*orange*), and cysteine (*yellow*) residues are highlighted for clarity.

Peptide F contains the cysteine residue that triggers the transition into amyloid, and it is preceded by two hydrophobic and rather unusual regions, a triple-methionine sequence (M51–M53, peptide E) and a tetra-leucine repeat (L62–L66, peptide F). These triple-methionine and tetra-leucine sequences are generally well conserved for p16 among vertebrate species (Fig. 7), and they could represent a novel amyloid-prone motif. Interestingly, individual residues of these motifs are fully solvent exposed in the monomeric structure (M53 and L65), thereby not contributing to the hydrophobic core of the monomer.

We previously reported the computationally predicted amyloid formation region to be in the range of residues 91 to 99 (3), corresponding to peptide G in this work. All four prediction algorithms favored this region, but the Aggrescan and Zyggregator programs also suggested the methionine and leucine repeat region (51–66) to be highly favorable to form amyloid (25, 26), which is in good accordance with our experimental findings. Further experiments dissecting the cysteine-containing peptide P1 revealed that the presence of three key regions lead to spontaneous aggregation: the triple-methionine region, the tetra-leucine region, and the site containing the cysteine residue. The presence of oxidant leads to both higher aggregation rates and a different morphology of the formed fibers, suggesting a significant role of dimeric building blocks, even in isolation.

No combined evidence of self-assembly was detected for the C-terminal region (P3) with the applied methods. Nevertheless, the limited digestion approach suggests protection from proteolysis for the peptides J and K, and fluorescence dye analysis showed a reproducible ThT signal increase of P3, which includes the computationally predicted amyloid region 91 to 99. When dissecting the peptide into fragments, some regions were able to form aggregates. This was also apparent from the electron microscopy data that showed amyloid-like structures for the peptide P3.2 (corresponding to peptide G) and aggregates for peptide P3.1 (corresponding to peptides I–K). Nevertheless, the ThT fluorescence and NMR signals did not change noticeably when freshly dissolved. It is likely that the weak amyloid formation propensity of sequences within P3 are enhanced in the context of the full protein sequence and when in proximity to other β-aggregation prone sequences.

The two identified sequences here are consistent with previously recorded solid-state NMR data (3), which identified the presence of leucine, valine, alanine, serine, and threonine residues in the β-sheet core, and the spectrum is also consistent with the presence of β-sheet methionine residues in the structured core.

Although the limited digestion approach is able to reveal differences between the two structural states of p16, it can only yield insights into the accessibility of specific cleavage sites. One other shortcoming of this method is the necessary assumption that the protein maintains structured regions upon partial cleavage (27, 28). Furthermore, the amyloid likely consists of several β-sheet–based protofilaments that are twisted into a fiber-like structure with variable protease accessibility. Nevertheless, the consistent differences in digestion rates provided us with a rational guide to design and test shorter peptides. These peptides were capable of self-assembly to various degrees and suggested the involvement of an N-terminal region containing Cys72, as well as a distal C-terminal region. It is possible that oxidation of the protein and subsequent dimerization impact the structure and expose these aggregation-prone regions for subsequent folding into amyloid. Therefore, this work sets the basis for further studies on the refolding of the dimer intermediate, for example, by introduction of mutations that alter their formation or kinetics.

## Experimental procedures

### Protein expression and purification

The DNA sequence encoding human p16 (UniProt ID P42771) was synthesized using *E. coli* optimized codons by GenScript Biotech Corporation harboring a 5′-NCO1 and a 3′-KPN1 restriction site. The DNA sequence was provided in a pET-based vector providing kanamycin resistance and an N-terminal hexa-histidine tag followed by a protein A sequence and a tobacco etch virus (TEV) cleavage site. This introduced an additional N-terminal glycine-alanine sequence after TEV cleavage and removal of the tag. The human sequence of p16 was expressed in *E. coli* BL21(DE3) cells. For this, cells were grown in either LB medium or minimal medium including ^15^NH_4_Cl as the sole nitrogen source at 25 °C overnight, followed by dilution to an *A*_600_ value of 0.6 and induction with 0.5 mM IPTG for 18 h at 19 °C. The protein was then purified by ultrasonication of the cells followed by clearing of the lysate by centrifugation for 1 h at 12,000 g and 4 °C. The supernatant was applied to Ni-NTA resin (Qiagen) using a gravity column set-up according to the manufacturer's instructions. The lysis and wash buffer consisted of 20 mM Hepes, 110 mM sodium acetate, 20 mM imidazole, and 2 mM β-mercaptoethanol at pH 8.0. The protein was eluted in wash buffer including 220 mM imidazole and applied to an ÄKTA purifier equipped with a HiLoad 16/600 Superdex 75 pg (Cytiva) running with wash buffer. The target protein peak was pooled and digested overnight with TEV protease at an approximately 1:50 TEV to protein ratio at 4 °C. The protein was then applied again to the Ni-NTA column to remove the cleaved N-terminal tag, any uncleaved protein, and the hexa-histidine-tagged TEV protease. The flow-through fraction was buffer exchanged into sample buffer (4 mM Hepes, pH 7.4) using the ÄKTA pure system equipped with a desalting column (HiPrep 26/10 Desalting, Cytiva) and, upon concentration to 30 μM, was stored in aliquots at −80 °C.

### p16 dimerization analysis

Time-dependent analysis of p16 dimerization was performed by non-reducing SDS-PAGE assays. Samples of 20 μM p16 were incubated with oxidant (diamide, 200 μM) at room temperature. After the indicated duration, remaining free cysteine was blocked with 10 mM NEM for 5 min to prevent artifactual oxidation and dimerization upon denaturation. Samples were then combined with non-reducing sample buffer and resolved on a 4 to 15% gradient Mini-Protean TGX Stain-Free gel (Bio-Rad) at 200 V. Molecular weights of resolved species were estimated by comparison to Precision Plus Protein Dual Color Standards (Bio-Rad).

### ThT fluorescence assays

Stock peptide solutions of 1 mg/ml (∼240 μM) were obtained by solubilization of peptides in a standard sample buffer (4 mM Hepes, pH 7.4). Immediately afterward, 80 μM peptide was combined with 10 μM ThT dye in a 96-well half area non-binding plate (Corning 3881). Where indicated, the oxidant diamide was also included in the reaction at 200 μM and total sample volume was 50 μl. The plate was sealed with a MicroAmp Optical Adhesive Film (Thermo Fisher Scientific) and measured on a Molecular Devices M5 microplate reader set to 25 °C. After an initial 10 s linear mixing step, ThT fluorescence was measured (Ex/Em 435/482 nm) at 2 min intervals under quiescent conditions in bottom read mode. Four replicates for each condition were measured, and these experiments were performed twice from independent peptide solutions.

### Dynamic scanning fluorimetry

The p16 protein samples were diluted to 20 μM, and a SYPRO Orange reporter dye was diluted to a 5x concentrate in the final measured samples. Five replicates were performed for each protein condition, including buffer controls. The samples were prepared and dispensed into a MicroAmp Optical 96-Well Reaction Plate (Applied Biosystems TM by Thermo Fisher Sc., N8010560) and covered using MicroAmp Optical Adhesive Film (Applied Biosystems TM by Thermo Fisher Sc., 4311971). The plate was then heated in a QuantStudio TM 3 Real-time PCR system at 0.15 °C/min until the temperature reached 95 °C, and data were analyzed using Protein Thermal Shift Software 1.4.

### Limited proteolysis

Samples contained 20 μM of either monomeric or amyloid p16 protein. Amyloid p16 was produced by addition of 200 μM diamide and subsequent incubation at room temperature for 16 to 17 h or 70 to 75 h, while the monomeric protein was kept at 4 °C for the corresponding length of time to ensure stability. NEM (10 mM) was added to the samples approximately 15 min prior to the addition of the protease in order to prevent artifactual disulfide bond formation. Limited digestion was then performed using either a 10:1, 50:1, or 100:1 substrate:trypsin protease (Promega) weight ratio with incubation at 25 °C for varying lengths of time (5 min to 24 h digestion times). Digestion was stopped by the addition of 0.1% formic acid.

When ^14^N-labeled p16 was mixed 1:1 with ^15^N-labeled p16, the samples were either digested in bulk, where samples were taken at each time point and mixed with 0.1% formic acid to stop the digestion reaction, or digested separately starting at different time points in small aliquots (25 μl) and stopped with formic acid simultaneously. The ratio of trypsin:protein used was 1:10, 1:50, or 1:100. The sample combinations alternated between isotopically labeled amyloid protein (^15^N-labeled) mixed with normal isotope abundance (^14^N-labeled) monomer protein and *vice versa*.

### Mass spectrometry

Samples were analyzed using a Thermo Scientific Velos Pro ion trap mass spectrometer coupled to a Dionex UltiMate 3000 HPLC system with a 50 μl injection loop (Thermo Scientific). Water containing 0.1% formic acid was used as Solvent A, and acetonitrile containing 0.1% formic acid was used as Solvent B. The samples were stored on an autosampler tray at 5 °C prior to injection. Nitrogen was used as the sheath gas, and the temperature of the heated capillary was 275 °C.

Digested protein samples (approximately 14 μg) were injected onto a Jupiter 4 μm Proteo 90 Å column (150 x 2.0 mm, Phenomenex) set to a temperature of 40 °C. The column was equilibrated with 95% Solvent A and 5% Solvent B for 5 min before a linear gradient was run for 20 min to 55% Solvent A and 45% Solvent B in order to achieve separation. The column was then flushed with 5% Solvent A and 95% Solvent B for 5 min and subsequently re-equilibrated in the initial conditions for 5 min. A flow rate of 0.2 ml/min was used throughout.

Data were analyzed using Thermo Xcalibur Qual Browser 4.2.47 programs (Thermo Fisher Scientific Inc.). The eluted tryptic peptides were analyzed using a data-dependent n^th^ order double play scan procedure. The 10 most abundant peaks in a full scan (300–2000 m/z) were sequentially selected, and an MS/MS scan was performed using collision-induced dissociation with a normalized collision energy of 40%. MS/MS spectra were recorded using dynamic exclusion with a repeat count of three, a repeat duration of 30 s, and an exclusion duration of 60 s. Fragmentation patterns were analyzed, and peptide fragment ions were assigned based on Roepstorff–Fohlman nomenclature (29).

For quantification of the tryptic peptides, peak areas were obtained by filtering the acquired mass spectra with the predicted m/z values of the expected charge states of each of the tryptic peptides (within the 300–2000 m/z range) and using Genesis peak detection (Gaussian 7-point smoothing). The identity of each of the detected peptides was confirmed by comparing the acquired corresponding MS/MS fragmentation patterns to the theoretical peptide fragment ions predicted using the mMass software (version 2.4).

### Negative-stain TEM

Samples for TEM analysis were prepared by incubating peptide suspensions (1 mg/ml) or full-length p16 protein (20 μM) in 4 mM Hepes, pH 7.4, on a carbon-coated copper grid (ProSciTech) for 60 s. Grids were then washed once with water and stained by incubating with uranyl acetate (2% w/v) for 30 s. Excess stain was removed using filter paper, and grids were left to dry overnight before measurement on a Philips CM-200 transmission electron microscope operating at 200 kV and equipped with a Gatan camera (Oxford Instruments).

### Solution NMR spectroscopy

Stock solutions of 1 mg/ml peptide were freshly prepared in 10 mM phosphate, pH 7.4 buffer and immediately diluted to 80 μM. ^2^H_2_O (9% v/v) was added for the lock signal leading to a total volume of 550 μl. Solution ^1^H NMR spectra were collected on a Bruker Avance III HD 600 MHz NMR spectrometer equipped with a 5 mm TXI triple-resonance probe including z-gradients. Spectra were acquired including an excitation sculpting solvent suppression scheme accumulating 256 scans at 298 K at the described time points between 15 min and 24 h. 32k complex data points were acquired, and an exponential window function of 2 Hz was applied to the free induction decay. Data were analyzed using Topspin 3.6.5.

### Fourier-transform infrared spectroscopy

Samples of 80 μM peptides P1-P3 in 10 mM phosphate buffer, pH 7.4, were dried using a SpeedVac Concentrator (Thermo). Spectra of the solid samples were recorded on a Bruker Alpha II FT-IR spectrometer with attenuated total reflectance attachment. Samples were subject to 128 scans at a resolution of 4 cm^−^^1^ at room temperature. Spectra were baseline corrected and smoothed using a Savitzky-Golan algorithm.

### Evolutionary analysis of amyloid prone region

The ENSEMBL database was used to obtain amino acid sequences of vertebrate orthologs of p16 (30). Ortholog alignment and visualization of the amyloid prone region of p16 were carried out using Jalview 2.11.2.7 software, with the Clustal plugin using default settings (31).

### Materials

Chemicals were purchased from Sigma Aldrich in the highest purity available unless stated otherwise. ^15^N ammonium chloride (99%) was purchased from Cambridge Isotopes Laboratories, Inc. Sequencing-grade modified trypsin was purchased from Promega. Truncated p16 regions P1-P3 were purchased from Mimotopes Pty Ltd (Australia) with a purity of at least 70% and peptides P4-P14 with a purity of at least 90%.

## Data availability

All data are contained within the manuscript and will be provided upon request.

## Supporting information

This article contains supporting information (14).

## Conflict of interest

The authors declare that they have no conflicts of interest with the contents of this article.
