# Supplementary material for: Characterizing the amyloid core region of the tumor suppressor protein p16INK4a using a limited proteolysis and peptide-based approach
Source: J Biol Chem. 2024 Jul 18;300(8):107590. doi: 10.1016/j.jbc.2024.107590 (PMC11375262; doi:10.1016/j.jbc.2024.107590)
Supplement: Supplementary data [file mmc1.docx]

**Supporting Information**

Characterizing of the amyloid core region of the tumor suppressor protein p16^INK4a^ using a limited proteolysis and peptide-based approach

Sarah G. Heath^1#^, Jennifer D. Naughton^1#^, Nicholas J. Magon^1^, Shelby G. Gray^2^, Briana R. Smith^1^, Vanessa K. Morris^2,3*^, Christoph Göbl^1,3^*

^1^ Mātai Hāora - Centre for Redox Biology and Medicine, Department of Pathology and Biomedical Science, University of Otago, Christchurch, New Zealand

^2^ School of Biological Sciences, University of Canterbury, Christchurch, New Zealand

^3^ Biomolecular Interaction Centre, University of Canterbury, Christchurch, New Zealand

^#^these authors contributed equally

*Vanessa K. Morris

Email: vanessa.morris@canterbury.ac.nz

*Christoph Göbl

Email: christoph.goebl@otago.ac.nz

Running title: Characterization of p16^INK4a^ amyloids

Keywords: amyloid, protein stability, protein oxidation, p16^INK4a^, mass spectrometry


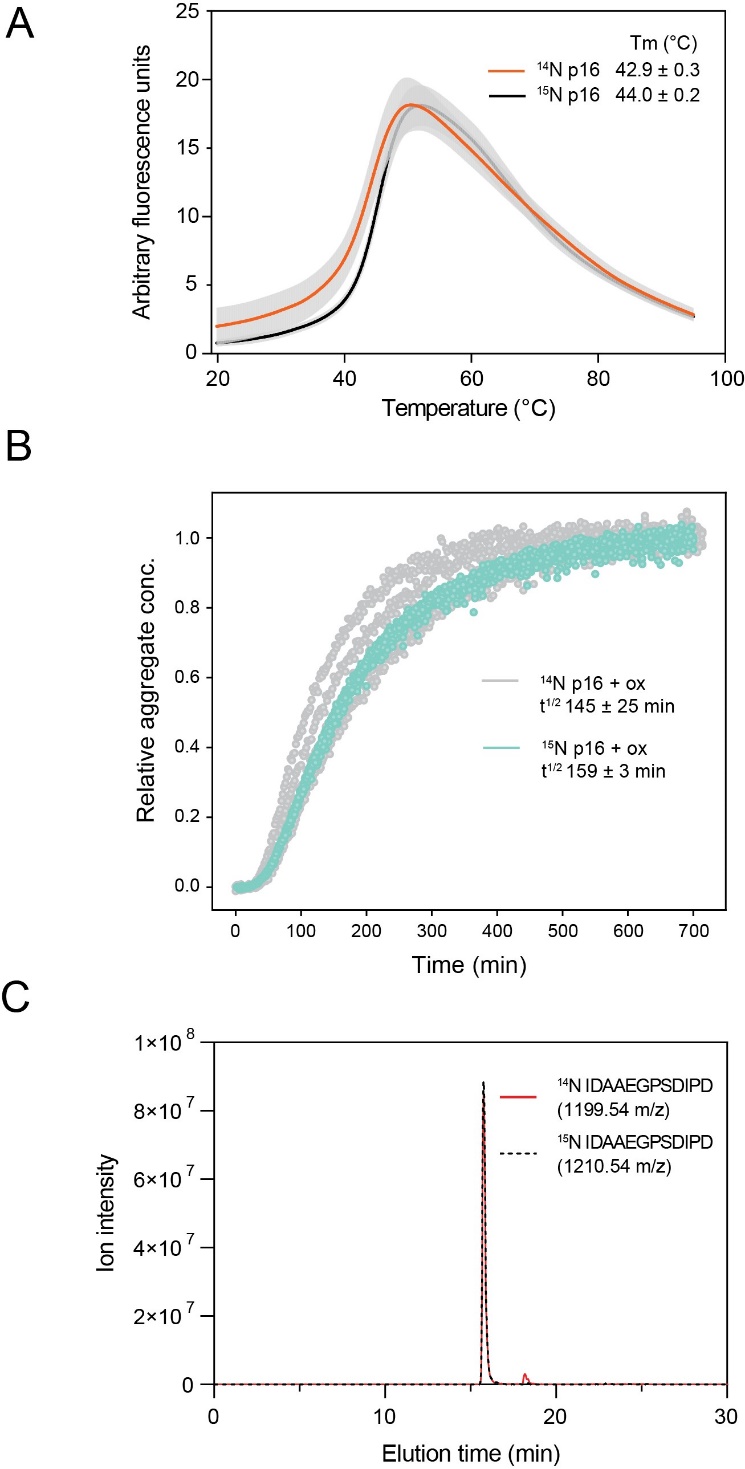


**Supplementary Figure 1.**

*(A)* Differential scanning fluorimetry (DSF) analysis of uniformly-labeled ^14^N and ^15^N p16 in the presence of Sypro Orange. Error bars in gray represent standard deviation from five measurements. Melting temperatures (T_m_) were derived from the maximum of the first derivative of the curves. *(B)* ThT aggregation assays of 20 μM samples of ^14^N and ^15^N labeled p16 in the presence of 200 μM diamide. Measurements were acquired in quadruplicate. Average half times of aggregation (t½) were calculated using the Amylofit platform [(17)](https://www.zotero.org/google-docs/?aqBMqh). *(C)* Representative overlaid selected ion chromatograms of peptide ‘P’ derived from uniformly-labeled ^14^N and ^15^N p16 upon digestion with trypsin for 24 hours and analyzed by LC/MS.


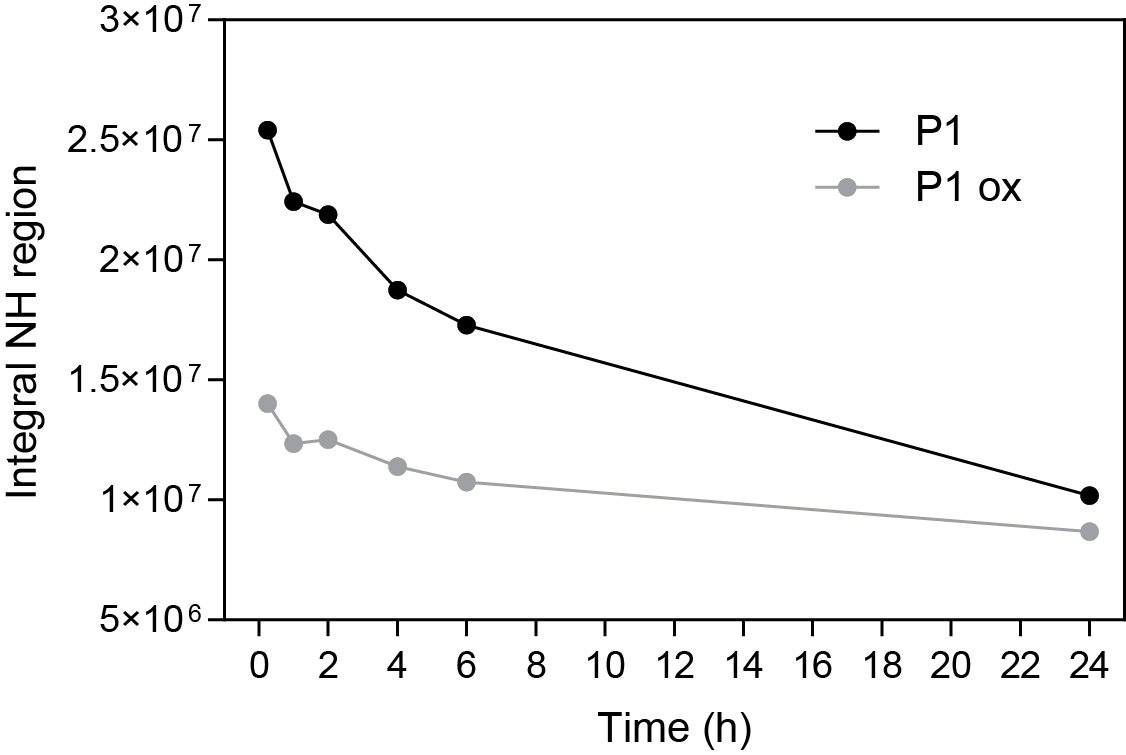


**Supplementary Figure 2.**

Quantification of NMR peak intensities from Fig. 4B derived from integration of the peak areas from 6.5 to 9.0 ppm (n=1).


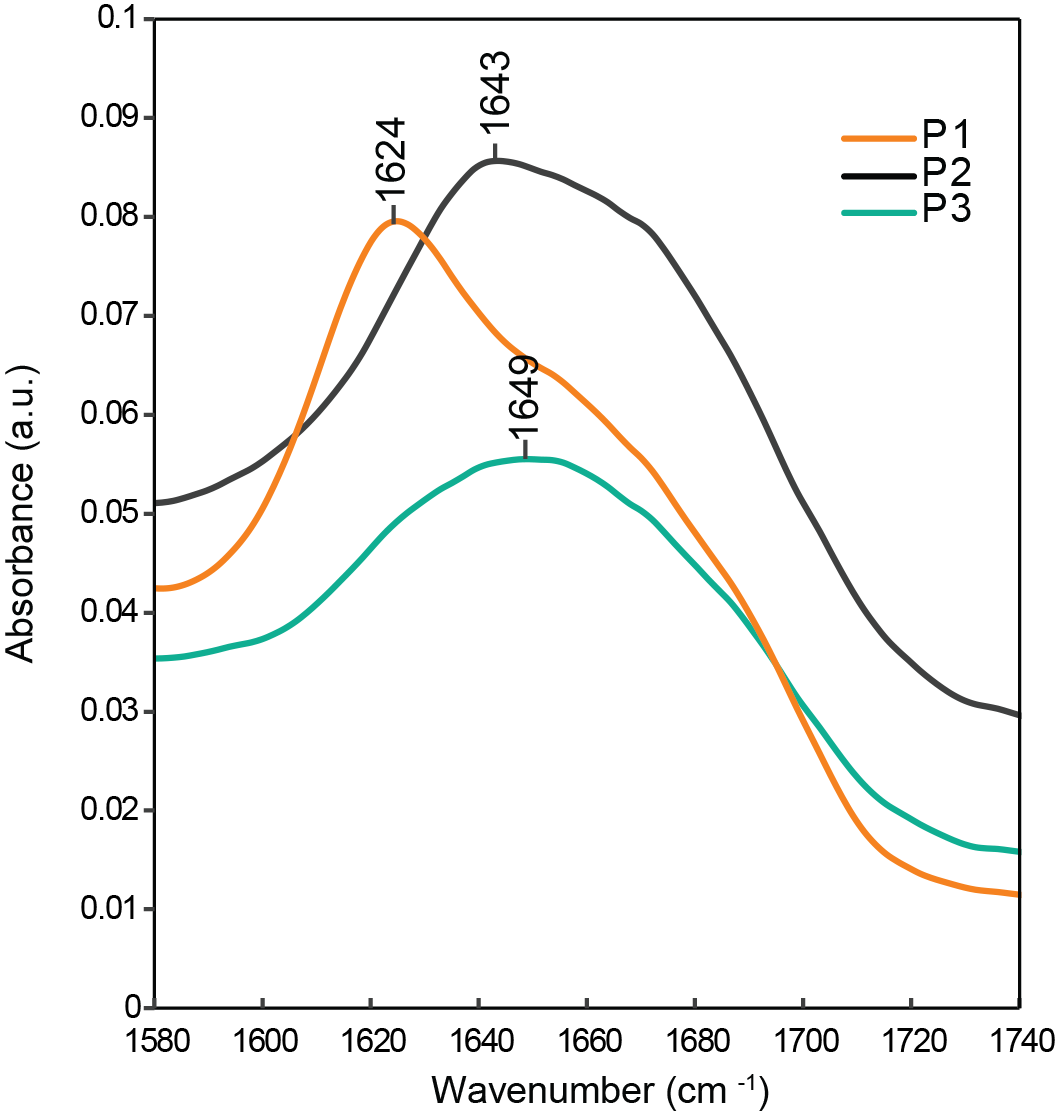


**Supplementary Figure 3.**

Fourier-transform infrared (FT-IR) spectroscopy measurements of the amide I region for peptides P1-P3. Samples of 80 μM peptide in 10 mM phosphate buffer, pH 7.4 were prepared and incubated at room temperature for 48 hours. Aliquots were dried to produce solid peptide samples which were measured (n=1) using a Bruker Alpha II FT-IR spectrometer.

**Supplementary Table 1.**

| Peptide | Position | Sequence | Retention time (min) | ^14^N m/z [M+H]^+1^ theoretical | ^14^N m/z [M+H]^+1^ observed | ^15^N m/z [M+H]^+1^ theoretical | ^15^N m/z [M+H]^+1^ observed |
| --- | --- | --- | --- | --- | --- | --- | --- |
| A | -2-22 | GAMEPAAGSSMEPSADWLATAAAR | 18.30 | 2348.08 | 2349.49 | 2376.08 | 2376.96 |
| B | 23-24 | GR | ND | 232.14 | ND | 237.14 | ND |
| C | 25-29 | VEEVR | 3.20 | 631.34 | 631.36 | 639.34 | 639.33 |
| D | 30-46 | ALLEAGALPNAPNSYGR | 16.60 | 1713.89 | 1714.18 | 1735.89 | 1735.36 |
| E | 47-58 | RPIQVMMMGSAR | 14.25 | 1376.70 | 1377.31 | 1395.7 | 1395.39 |
| F-NEM | 59-87 | VAELLLLHGAEPN**C**ADPATLTRPVHDAAR | 17.25 | 3177.57* | 3177.50 | 3217.57* | 3216.95 |
| F-F | (59-87)2 | (VAELLLLHGAEPN**C**ADPATLTRPVHDAAR)2 | 17.30 | 6103.90* | 6102.68 | 6183.9* | 6180.75 |
| G | 88-99 | EGFLDTLVVLHR | 19.10 | 1398.77 | 1399.12 | 1415.77 | 1415.31 |
| H | 100-103 | AGAR | ND | 374.22 | ND | 381.77 | ND |
| I | 104-107 | LDVR | 3.20 | 502.30 | 502.23 | 509.30 | 509.30 |
| J | 108-112 | DAWGR | 4.85 | 604.28 | 604.22 | 613.25 | 613.28 |
| K | 113-124 | LPVDLAEELGHR | 16.50 | 1348.72 | 1349.23 | 1365.72 | 1365.45 |
| L | 125-128 | DVAR | ND | 460.25 | ND | 467.25 | ND |
| M | 129-131 | YLR | 3.15 | 451.27 | 451.22 | 457.27 | 457.32 |
| N | 132-138 | AAAGGTR | ND | 603.32 | ND | 613.32 | ND |
| O | 139-144 | GSNHAR | ND | 641.31 | ND | 653.31 | ND |
| P | 145-156 | IDAAEGPSDIPD | 15.8 | 1199.54 | 1199.49 | 1211.54 | 1211.12 |

Experimental data missing on undetected peptides marked ND (not detected).
*Listed peptide mass-to-charge (m/z) values are monoisotopic except in the case of peptide F where the average peptide mass-to-charge ratios are reported.
